# Acute impact of inorganic nitrate supplementation after ischemia and during small muscle mass exercise in postmenopausal females: A pilot study

**DOI:** 10.14814/phy2.70076

**Published:** 2024-10-04

**Authors:** Jacob T. Caldwell, Alyssa Koenke, Lauren Zimmerman, Aaron E. Wahl, Sarah A. Fenn, Emily E. Grammer, Macy E. Stahl, Jason D. Allen, Salvador J. Jaime

**Affiliations:** ^1^ Exercise and Sport Science Department University of Wisconsin‐La Crosse La Crosse Wisconsin USA; ^2^ Department of Kinesiology, School of Education and Human Development University of Virginia Charlottesville Virginia USA; ^3^ Division of Cardiovascular Medicine, School of Medicine University of Virginia Charlottesville Virginia USA

**Keywords:** aging, blood flow, ischemic exercise, near‐infrared spectroscopy, oxygenation

## Abstract

Menopause is associated with reduced endothelial‐dependent vasodilation and increased cardiovascular disease (CVD) risk. Dietary nitrate, a non‐pharmacological approach, may increase vasodilatory capacity consequentially reducing CVD risk. We investigated macro‐ and microvascular function after acute nitrate supplementation in postmenopausal females (PMF). Vascular function was studied with flow‐mediated vasodilation (FMD) and near‐infrared post occlusive reactive hyperemia (PORH). Incremental handgrip exercise was performed to investigate blood flow and tissue oxygenation. We hypothesized acute dietary nitrate would not impact resting endothelial measures but would increase post ischemic vasodilation and incremental exercise blood flow. Late‐phase PMF (*n* = 12) participated in a randomized crossover design with 140 mL of nitrate‐rich (NR) beetroot juice or nitrate‐poor black currant juice. Testing included a 5‐min FMD, a 3‐min ischemic exercise FMD, and incremental exercise at 10%, 15%, and 20% maximal voluntary contraction to measure blood flow and pressure responses. A *p* ≤ 0.05 was considered significant. One‐way ANOVA indicated lower resting pressures, but no change to FMD, or PORH in either protocol. Two‐way repeated measures ANOVA indicated NR supplementation significantly reduced mean arterial pressure at rest and during incremental exercise at all intensities without changes to blood flow. Acute nitrate is effective for resting and exercising blood pressure management in PMF.

## 1 INTRODUCTION

Vascular endothelial function plays a crucial role in preventing cardiovascular (CV) disease via nitric oxide (NO) synthesis given its vasodilatory, anti‐inflammatory, and antithrombotic effects (Zhao et al., 2015). Estrogen modulates endothelial NO production, primarily through the endothelial NO synthase (eNOS) pathway (Anagnostis et al., 2022; Moreau et al., 2024; Rossouw, 2008; Rossouw et al., 2013). Postmenopausal females (PMF), however, have reduced estrogen levels which contribute to lower endothelial‐dependent vasodilation, blood flow, and vascular compliance (Anagnostis et al., 2022; Lakatta & Levy, 2003; Moreau et al., 2003; Nair et al., 2021). This is important given the vascular endothelial environment accounts for ~40% of CV risk reduction beyond traditional measures (i.e., blood pressure, body mass, lipids) (Green et al., 2008, 2014; Joyner & Green, 2009). Moreover, reductions in endothelial‐dependent vasodilation may elucidate the augmented central and peripheral pressures observed in PMF (Kim et al., 2019; Lakatta & Levy, 2003; Moreau et al., 2024), which result in heightened risk for CV disease and event risk (Moreau et al., 2020; Nair et al., 2021; Rossouw et al., 2013). While supplemental estradiol can increase endothelial‐dependent vasodilation (Moreau et al., 2020), its use is limited given the suboptimal effects on primary prevention of CV disease risk, increased risk of stroke and venous thromboembolism, opening avenues for supplemental approaches (Anagnostis et al., 2022; Nair et al., 2021).

Aerobic exercise training improves traditional CV risk factors (Moreau & Ozemek, 2017; Nair et al., 2021; Rossouw et al., 2013). However, it does not consistently enhance vascular endothelial health in PMF (Moreau et al., 2013; Moreau & Ozemek, 2017). Specifically, reduced estrogen levels, decreased availability of NO, and increased oxidative stress in PMF are believed to impair vascular endothelial function and limit adaptive responses to exercise training (Moreau et al., 2013). Despite PMF being resistant to endothelial‐training adaptations (Moreau et al., 2013), recent evidence indicates that a high‐intensity cycling session combined with a nitrate‐rich (NR) supplement can improve brachial artery flow‐mediated dilation (FMD) in PMF (Hogwood et al., 2023). These findings show that acute increases in bioavailable NO can enhance vascular endothelial health in PMF under specific exercise conditions (i.e., acidic and/or hypoxic) (Caldwell et al., 2019; Jones et al., 2018). However, the effects of acute nitrate supplementation on upper extremity macro‐ and microvascular function following an ischemic challenge (high intensity) or during incremental exercise remain underexplored in PMF (Caldwell et al., 2019; Moreau et al., 2003). Our study builds on previous research by employing a comprehensive testing battery before and during hand‐grip exercise to gain additional insights into vascular control at rest and during exercise in PMF through the acute enhancement of bioavailable NO with dietary nitrate.

The purpose of this investigation was to acutely increase bioavailable NO and evaluate its effects on resting and ischemic exercise‐induced brachial artery FMD, as well as post occlusive reactive hyperemia (PORH) using near‐infrared spectroscopy (NIRS). We also examined brachial artery blood flow and forearm microvascular saturation changes during incremental exercise. Moreover, this study aimed to replicate the improvements in central hemodynamic and peripheral blood pressure parameters previously reported (Hughes et al., 2016; Kim et al., 2019). We hypothesized that acute nitrate supplementation would not enhance resting FMD or PORH, but both would be increased during the ischemic exercise (Lundberg et al., 2018). Additionally, we anticipated that acute dietary nitrate would increase brachial artery blood flow and NIRS‐derived microvascular tissue oxygenation during incremental exercise and lower resting blood pressure (Caldwell et al., 2019; Ferguson et al., 2016; Jones et al., 2016). These findings are expected to advance our understanding of vascular control and functional impacts of acute dietary nitrate supplementation in PMF.

## 2 METHODS

### 2.1 Participants

Fourteen PMF [age 64 ± 1 years (mean ± SE); height 165 ± 1 cm; mass 73 ± 6 kg; body mass index 30 ± 5 kg/m^2^], volunteered to participate in the current investigation (Table 1). Criteria used for PMF were at least 1 year without a menstrual cycle and no cardiovascular disease (CVD), no hypertension medication, non‐smokers, no hormone therapy, or proton pump inhibitors. Of the 16 originally recruited, two females were excluded who were later diagnosed with hypertension and two were excluded as they withdrew from the study after the first visit due to pain from the 5‐min occlusion test. Two females were on a statin and four were on thyroid medication and continued taking as prescribed throughout the two‐week investigation. Females were classified as post‐menopausal based on answering questions from the health history forms indicating at least 1 year from menstruation and were considered “late phase” if it had been at least 6 years from their last full cycle based on previous work (Caldwell et al., 2019). All experimental procedures and methods were approved by the Institutional Review Board of the University of Wisconsin‐La Crosse and conformed to the standards set forth by the *Declaration of Helsinki*. Written informed consent and health history screening for overt diseases (e.g., cardiovascular, metabolic, renal) took place prior to data collection. All testing was completed in a temperature‐controlled laboratory (20–22°C). Blood was not taken from one female subject due to fear of needles and an *n* = 11 was used for nitrate and nitrite analysis.

**TABLE 1:** Participant baseline characteristics (*n* = 12). Only participants who completed the study are shown in this table.

Variable	
*n*	12
Age, year	64 ± 5
BMI, kg/m^2^	26 ± 6
Height, cm	164 ± 5
Mass, kg	68 ± 21
Cholesterol	*n* = 2
Thyroid	*n* = 4

### 2.2 Experimental protocol

The current investigation was a randomized, double‐blind, crossover study design separated by a 48‐h wash‐out period. Participants were randomly assigned to either a NR or nitrate‐poor (NP) supplement in the form of beetroot juice and black currant, respectively. Two‐hours before testing sessions, participants orally consumed either 140 mL of a concentrated beetroot juice supplement (BEET IT Sport, James White Drinks Ltd., Ipswich, UK) containing [~12.9 mmol NO_3_^−^], or NP placebo [<0.2 mmol NO_3_^−^], (Jungle Powders, Estonia). All participants were instructed to abstain from using mouthwash, maintain their regular diet, and be at least 4 h fasted.

During the first lab visit height and mass of each participant were taken followed by a blood draw for subsequent nitrate/nitrite analysis (Caldwell et al., 2019; Ferguson et al., 2013a). Next, participants were instructed to lay supine to obtain maximal voluntary contraction (MVC) of the right forearm by extending the right arm at heart level and to familiarize them with the protocol. Participants were instructed to exert maximal force on a handgrip dynamometer for 2 s with 1‐min rest intervals in triplicate. MVC was calculated based on an average of the two highest values and was used to calculate the following percentages for the ischemic and incremental exercise tests: 10%, 15%, and 20% MVC. Participants then rested quietly for 20 min followed by measurement of pulse wave analysis (PWA) and pulse wave velocity (PWV) in duplicate using the SphygmoCor XCEL (Atcor Medical, Sydney, AU).

**FIGURE 1:**
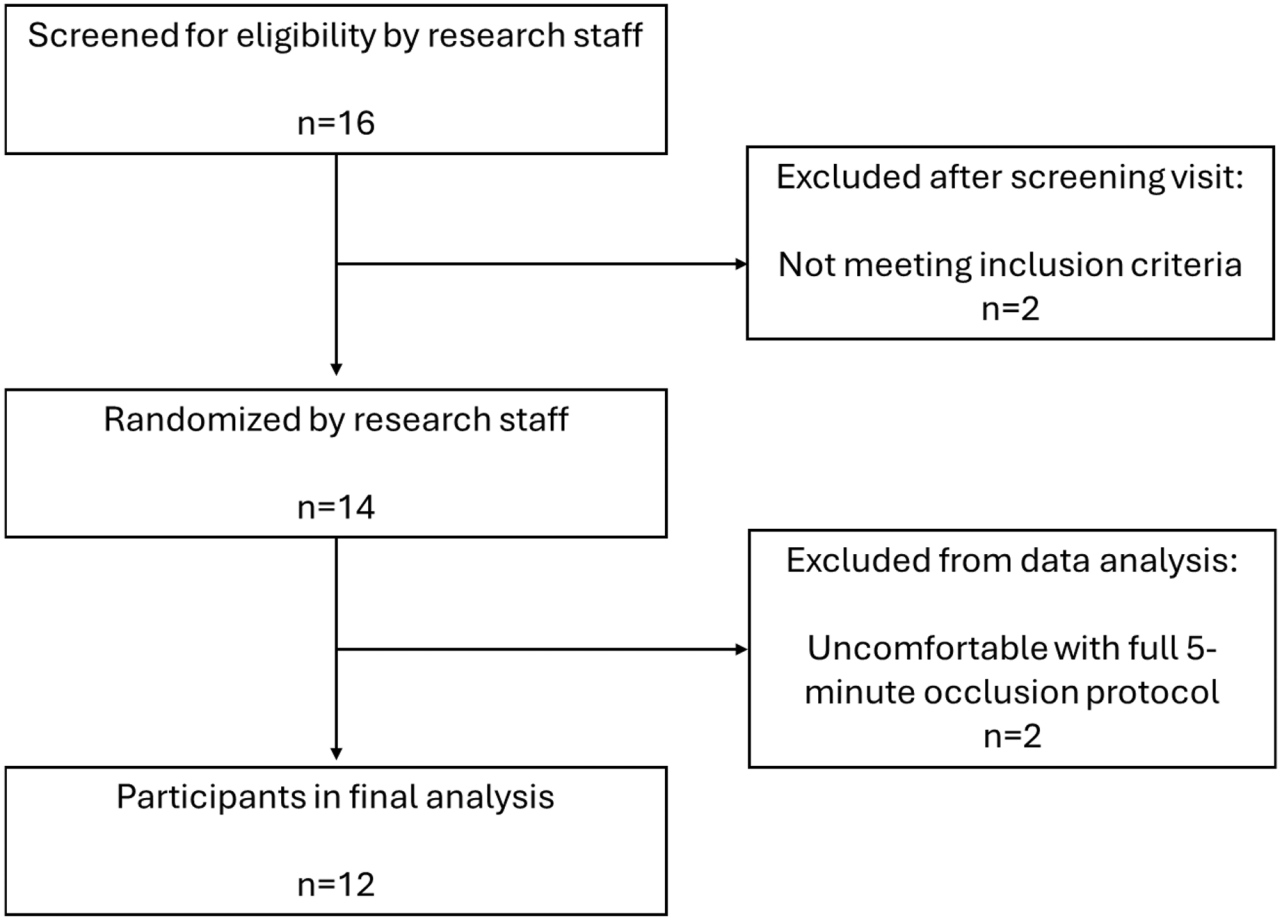
CONSORT Diagram.

**FIGURE 2:**
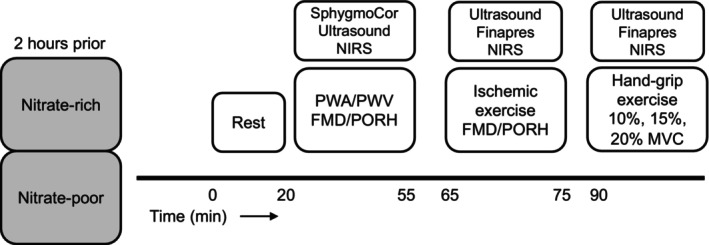
Study overview. PWA, pulse wave analysis; PWV, pulse wave velocity; FMD, flow‐mediated vasodilation; PORH, post occlusive reactive hyperemia. Not included in figure—on Day 1 MVC was performed prior to the 20‐min resting period.

Macrovascular brachial artery FMD and microvascular NIRS PORH of the right arm were measured next (Barstow, 2019; Caldwell et al., 2023). We first performed a traditional, primarily NO mediated (Thijssen et al., 2019; Thijssen & Green, 2020) brachial artery FMD with simultaneous NIRS PORH on the flexors of the right forearm by a 5‐min occlusion (Thijssen & Green, 2020). This was followed by 10 min of rest and then participants performed 3 min of ischemic exercise at 20% MVC to assess large vessel vasodilation and microvascular reperfusion immediately after cuff deflation (Figure 1). Ischemia was performed with the rapid inflation Hokanson cuff to 250 mmHg. Next, after at least a 15‐min break, participants performed incremental hand‐grip exercise. Handgrip exercise was performed on a custom‐built hand‐grip dynamometer in the right arm at a rate of 20 contractions per min at 10, 15, and 20% MVC for 3‐min per stage to obtain steady‐state measurements. On the second day, the same measures and time points were performed without the MVC (Figure 2).

## 3 EXPERIMENTAL MEASUREMENTS

### 3.1 Blood sampling

Venous blood samples were drawn at rest and placed into lithium heparin vacutainers (Becton Dickenson, NJ) centrifuged at 1500 RCF at 4°C for 10 min and immediately stored at −80°c for later analysis. Plasma NO_3_^−^ and NO_2_^−^ were assessed via ozone‐based chemiluminescence using a Sievers NOA model 280i (GE Analytical Instruments, Boulder, CO, USA) as previously described (Ferguson et al., 2013b). Briefly, plasma samples for NO_3_^−^ analysis were deproteinized using cold ethanol precipitation in a 1:3 dilution (plasma: ethanol) followed by a 30‐min incubation before being centrifuged at 14,000 *g* for 10 min. The supernatant was removed for the subsequent NO_3_^−^ analysis in the presence of vanadium chloride in hydrochloric acid at 95°C. The NO_2_^−^ of the undiluted (non‐deproteinized) plasma samples was determined by its reduction to NO in the presence of glacial acetic acid and potassium iodide as previously explained (Kenjale et al., 2011).

### 3.2 Cardiovascular measures

PWA was measured using the SphygmoCor XCEL using a blood pressure cuff placed on the upper left arm (Table 2). The device recorded 10 s of waveforms and repeated in duplicate if quality control was passed. SphygmoCor waveforms are composed of a pressure wave generated from the cardiac stroke volume, and the reflected wave that returns to the aorta from peripheral arteries (Nichols & Singh, 2002). Aortic and brachial blood pressures and other central indices such as the subendocardial viability ratio (SEVR) (Butlin & Qasem, 2017), augmented pressure (AP; difference between two systolic peaks), augmentation index (AIx) and AIx standardized at a HR of 75 bpm (AIx@75), expressed as ratios of arterial pressure and pulse pressure (Jaime et al., 2022) were used. Pressures of forward (Pf) and backwards (Pb) waves were calculated using a validated wave separation analysis (Westerhof et al., 2006).

**TABLE 2:** Effect of dietary nitrate on central and peripheral pressures and hemodynamics (*n* = 12 for all).

Variable	NR	NP	*p*‐value
PWV, m/s	6.9 ± 0.8	7.4 ± 0.8	0.079
Heart rate, BPM	61 ± 6	59 ± 6	**0.027**
Aortic systolic blood pressure, mmHg	112 ± 11	118 ± 10	**0.005**
Aortic diastolic blood pressure, mmHg	73 ± 10	75 ± 9	0.069
Aortic pulse pressure, mmHg	38 ± 7	43 ± 7	**0.01**
Brachial systolic blood pressure, mmHg	120 ± 12	127 ± 12	**0.002**
Brachial diastolic blood pressure, mmHg	72 ± 10	74 ± 9	0.11
Brachial pulse pressure, mmHg	48 ± 8	53 ± 8	**0.004**
Mean arterial pressure, mmHg	87 ± 10	90 ± 9	**0.022**
AIx	34 ± 6	33 ± 9	0.32
AIx@75	27 ± 10	27 ± 8	0.66
Pf	152 ± 17	159 ± 22	0.23
Pb	267 ± 13	278 ± 7	**0.002**
Forward pulse height, mmHg	25 ± 6	27 ± 4	0.20
Reflected pulse height, mmHg	16 ± 2	18 ± 3	0.09
Subendocardial variability ratio (%)	152 ± 25	141 ± 18	**0.003**

*Note*: Bold text indicates significant differences between treatments. Data are means ± SD.

Abbreviations: AIx, augmentation index; AIx@75, augmentation pressure corrected for heart rate 75; Pb, reflected pressure transit time; Pf, forward pressure transit time; PWV, carotid‐femoral pulse wave velocity.

PWV was measured next using the SphygmoCor XCEL. A blood pressure cuff was positioned on the left thigh distal to the bifurcation of the femoral artery and a high‐fidelity pencillike tonometer (Miller Instruments, Texas, USA) on the left common carotid artery. Care was taken to measure the thigh cuff distance from the patella during each visit to ensure repeatability and the carotid‐femoral PWV. The segments required to determine carotic‐femoral distance (carotid‐suprasternal notch, suprasternal notch‐femoral cuff, and femoral artery‐femoral cuff) were measured using a linear segmometer do avoid potential abdominal curvature which may impact the measurement. cfPWV was calculated by dividing the measured distance from the carotid and femoral artery by the pulse transit time between the two positions following the collection of 10 waveforms and corrected by a factor of 0.80 (Figueroa et al., 2020; Jaime et al., 2022). All measurements were taken in duplicate and reported as average.

After completion of PWA/PWV measurement, beat‐by‐beat HR, mean arterial (MAP), systolic (SBP) and diastolic blood pressure (DBP), were continuously measured for the remaining testing via finger photoplethysmography (Finometer Pro, FMS, The Netherlands), calibrated to brachial artery blood pressure according to manufacturer specifications.

### 3.3 Flow‐mediated vasodilation

Measurements of brachial artery diameter and blood velocity were simultaneously measured with an ultrasound system (Terason t3200, Burlington, MA) equipped with a multi‐frequency linear array transducer operating at 5 MHz, placed with care taken to avoid the bifurcation of the artery. All measurements had a Doppler sample volume set at the full width of the vessel with the insonation angle <60°. A rapid inflation and deflation pneumatic cuff (D.E. Hokanson, Bellevue, WA) was positioned just proximal to the elbow and distal to the ultrasound probe. Cuff pressure was increased to 75 mmHg above systolic pressure to create complete occlusion to the arm which was confirmed with palpation. Brachial artery images were captured for 5 min after cuff deflation for each protocol to insure peak diameter was reached and stored with Debut Professional video capture (NCH software) and diameters were analyzed offline using a commercially available edge‐detection and doppler flow analysis software package in line with manufacturer specifications (Quipu, Cardiovascular Suite 4, Italy) (Caldwell et al., 2023).

### 3.4 Brachial artery blood flow at rest and during exercise

Forearm blood flow (FBF) was measured in the same location as the FMD and was calculated as: FBF = mean blood velocity × 60 × π × (brachial diameter/2)^2^ calculated in mL min. Mean arterial blood pressure (MAP) was time aligned with FBF to calculate forearm vascular conductance (FVC) calculated in mL · min · 100 mmHg [FVC = (FBF / MAP) * 100] (Jendzjowsky & Delorey, 2013; Tschakovsky et al., 2002). All diameters, velocities and blood pressures were averaged for the final 30 s at each exercise intensity (i.e., min 2.5–3.0). See below for NIRS methods during exercise.

### 3.5 Near infrared spectroscopy

NIRS derived microvascular hemoglobin + myoglobin [Heme] was measured with a continuous wave multi‐distance NIRS probe (Portamon, Artinis, The Netherlands) that was placed longitudinally over the right forearm and secured with a flexible bandage and black cloth. The NIRS probe consists of a detector fiber bundle, three light‐emitting diodes (LED), and operates at wavelengths of 690 and 830 nm (source detector distance 2.5–4.0 cm). The NIRS device allows for relative changes in oxygenated (oxy‐[Heme]), deoxygenated (deoxy‐[Heme]) and the calculated sum, total (total‐[Heme]). The use of [Heme] is preferred as the NIRS cannot distinguish between hemoglobin or myoglobin (Barstow, 2019). The present work uses tissue saturation (StO_2_) when discussing muscle saturation, which is calculated as oxy‐[Heme] divided by total‐[Heme]. The NIRS probe was allowed to run for at least 2‐min prior to the start of data collection. The muscle belly was identified by a single experienced investigator palpating during muscle contraction, remaining in position throughout testing and marked with a surgical marker to reproduce placement 1 week following. The NIRS PORH parameters were determined using the following methods: Baseline percent saturation was averaged over the minute before cuff inflation. The desaturation rate was measured as the downward slope from 10 to 60 s after cuff inflation. The tissue resaturation rate was determined by the upward slope in the 10‐s window following cuff release. The nadir was identified as the minimum value during occlusion. The peak was identified as the maximum value after cuff release. The area under the curve (AUC) was calculated using the trapezoid rule during occlusion and after cuff deflation (Barstow, 2019; McLay, Fontana, et al., 2016; McLay, Gilbertson, et al., 2016). During incremental exercise, NIRS tissue oxygenation was assessed as the average of the final 30‐s of each stage and compared between stages. The NIRS data were collected throughout the study protocol at 10 Hz and stored for post hoc analysis in Microsoft Excel.

### 3.6 Statistics

Data were analyzed with commercially available statistical software package (Sigmaplot; version 14.5, Systat software, San Jose). A one‐way (treatment group) analysis of variance (ANOVA) was used to analyze resting CV function at least 2‐h after each supplement with the SphygmoCor XCEL (Table 2). A one‐way (treatment group) ANOVA was also used for FMD and NIRS derived analysis (Table 3) All exercise data were analyzed with a two‐way (time × treatment group) repeated measures ANOVA with Student–Newman–Keuls post hoc test for pairwise comparison. The level of significance was set at (*p* < 0.05). All data are presented as means ± standard deviation.

**TABLE 3:** Effect of dietary nitrate on micro‐ and macrovascular parameters (*n* = 12 for all).

	NR	NP	*p*‐value
Near‐infrared spectroscopy variables			
Traditional PORH			
Baseline, %	65 ± 4	65 ± 3	0.99
Nadir, %	33 ± 8	34 ± 7	0.6
Peak, %	76 ± 2	77 ± 3	0.21
Recovery, %	66 ± 4	66 ± 4	0.95
Occlusion, AUC	145,223 ± 19,397	147,391 ± 15,062	0.68
Slope 1, %/s	−0.10 ± 0.03	−0.10 ± 0.02	0.91
Ischemic PORH			
Baseline, %	67 ± 4	67 ± 4	0.71
Nadir, %	27 ± 7	27 ± 7	0.77
Peak, %	74 ± 4	75 ± 2	0.19
Recovery, %	72 ± 5	73 ± 5	0.6
Occlusion, AUC	63,030 ± 15,377	65,656 ± 12,318	0.55
Slope 1, %/s	−0.63 ± 0.8	−0.46 ± 0.6	0.61
Flow‐mediated vasodilation variables			
Traditional FMD			
Baseline diameter, mm	3.45 ± 0.51	3.48 ± 0.49	0.91
Maximal diameter, mm	3.75 ± 0.57	3.73 ± 0.56	0.5
Normalized FMD, %FMD/SS_AUC_	4.63E‐04 ± 1.33E‐04	3.63E‐04 ± 1.02E‐04	0.08
Shear AUC, AU	23,031 ± 9470	22,400 ± 5365	0.72
Ischemic FMD			
Baseline diameter, mm	3.45 ± 0.44	3.38 ± 0.57	0.59
Maximal diameter, mm	3.77 ± 0.51	3.72 ± 0.61	0.63
Normalized FMD, %FMD/SS_AUC_	2.98E‐04 ± 2.00E‐04	1.86E‐04 ± 1.00E‐04	0.13
Shear AUC, AU	39,283 ± 14,612	61,866 ± 22,702	**0.008**

*Note*: Top of the table represents baseline NIRS variables during the traditional 5‐min occlusion and 3 min of ischemic exercise. Bottom of the table represents brachial artery responses to traditional and ischemic exercise. *p‐*value represents one‐way ANOVA. Bold text indicates significant differences between treatments. Data are means ± SD.

Abbreviations: FMD, flow‐mediated vasodilation; PORH, post occlusive reactive hyperemia.

## 4 RESULTS

**FIGURE 3:**
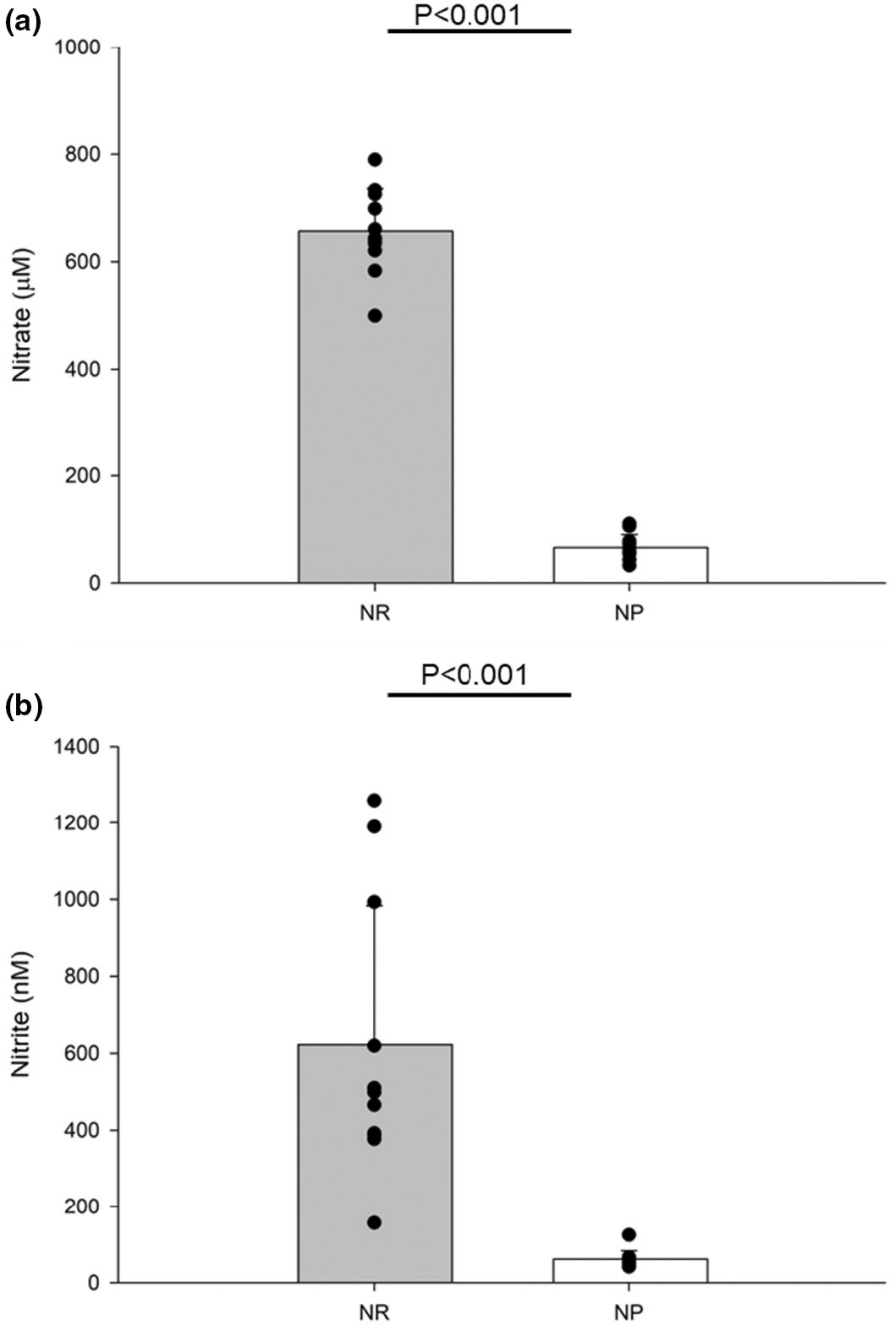
(a) Plasma nitrate levels between treatment of a nitrate rich (*n* = 11) or poor supplement (*n* = 11), and (b) plasma nitrite levels between treatment of a nitrate rich (*n* = 11) or nitrate poor supplement (*n* = 11). Data were run with one‐way ANOVA. Data are means ± SD.

**FIGURE 4:**
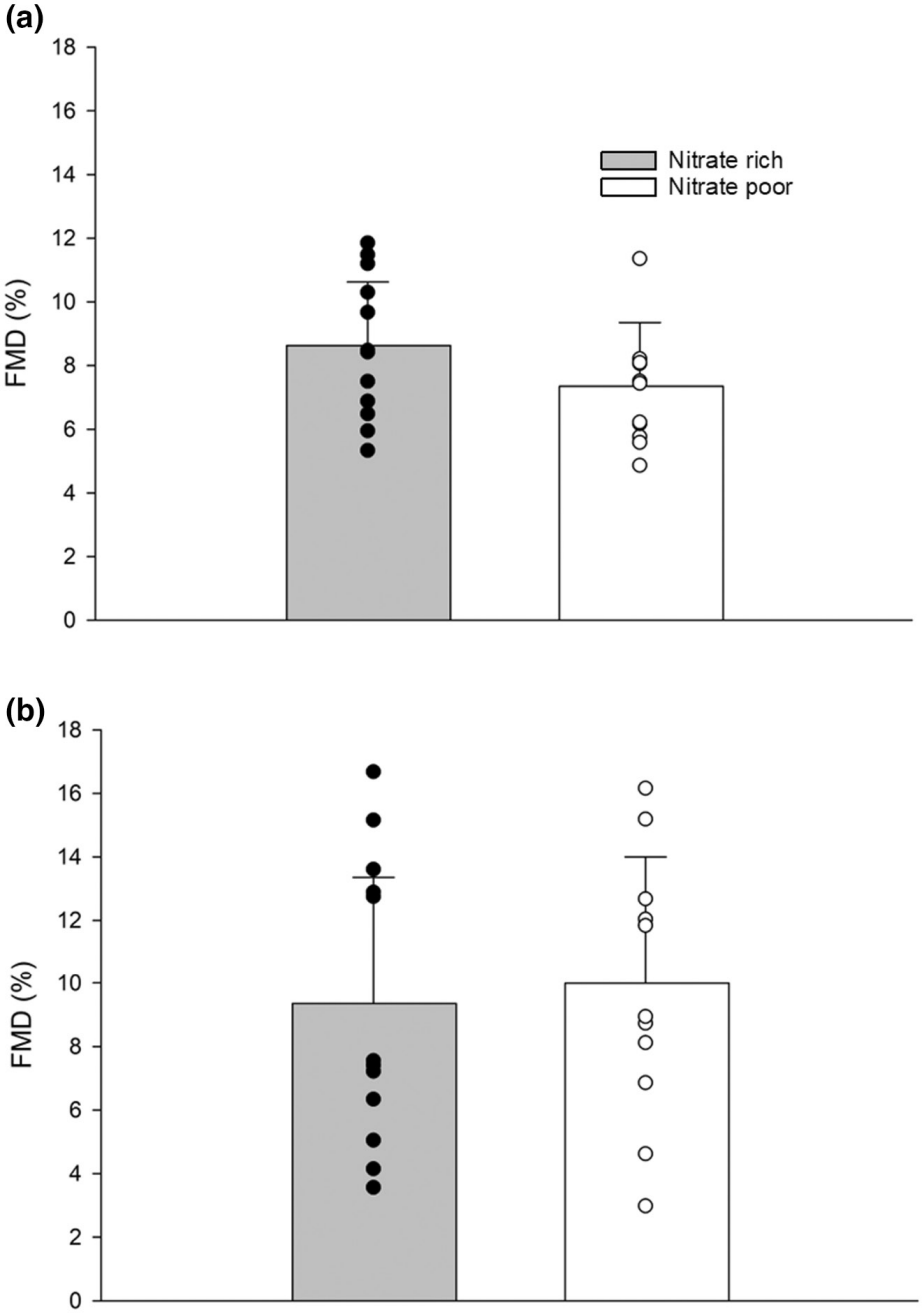
(a) No significant change in brachial artery flow‐mediated vasodilation during a traditional 5‐min occlusion at rest between nitrate rich (*n* = 12) and nitrate poor conditions (*n* = 12). (b) No significant change in brachial artery flow‐mediated vasodilation after 3 min of ischemic handgrip exercise at 20% MVC between nitrate rich (*n* = 12) and nitrate poor (*n* = 12) conditions. Data were run with one‐way ANOVA. Data are means ± SD.

**FIGURE 5:**
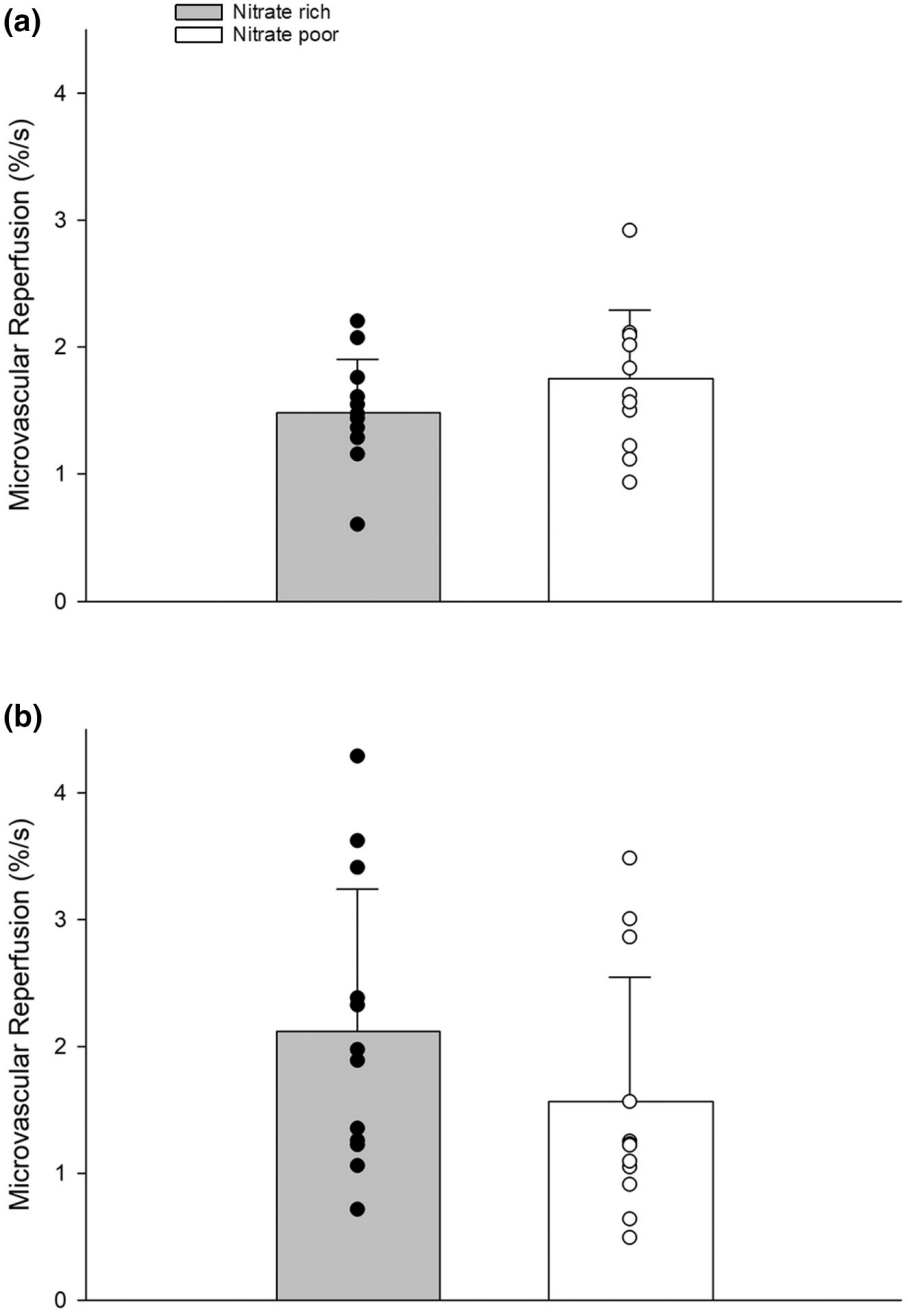
(a) Forearm near‐infrared spectroscopy microvascular reperfusion (StO_2_%/second) during a traditional 5‐min occlusion at rest between nitrate rich (*n* = 12) and nitrate poor conditions (*n* = 12). (b) Forearm NIRS microvascular reperfusion after 3 min of ischemic handgrip exercise at 20% MVC between nitrate rich (*n* = 12) and nitrate poor (*n* = 12) conditions (*p* > 0.05). Data were run with one‐way ANOVA. Data are means ± SD.

Acute dietary nitrate supplementation increased plasma nitrate and nitrite (Figure 3). Acute supplementation did not change resting or ischemic exercise FMD (Figure 4). Further, no changes were seen with resting or ischemic exercise PORH (Figure 5). We compared FMD and PORH responses during a traditional resting five‐minute, and a 3‐min ischemic hand‐grip exercise bout. Baseline diameters during traditional FMD (NR, 3.45 ± 0.18; NP, 3.48 ± 0.17%) and ischemic exercise FMD (NR, 3.45 ± 0.12; NP, 3.38 ± 0.16%) were not significantly different between treatments. FMD was not significantly higher in the NR condition for the traditional (NR, 8.6 ± 0.83; NP, 7.3 ± 0.71%) or ischemic (NR, 9.3 ± 1.31; NP, 10.0 ± 1.15%) exercise FMD. Shear stress did not play a factor in either condition as normalized FMD to shear stress was also not different (*p* > 0.05) (Table 3).

**FIGURE 6:**
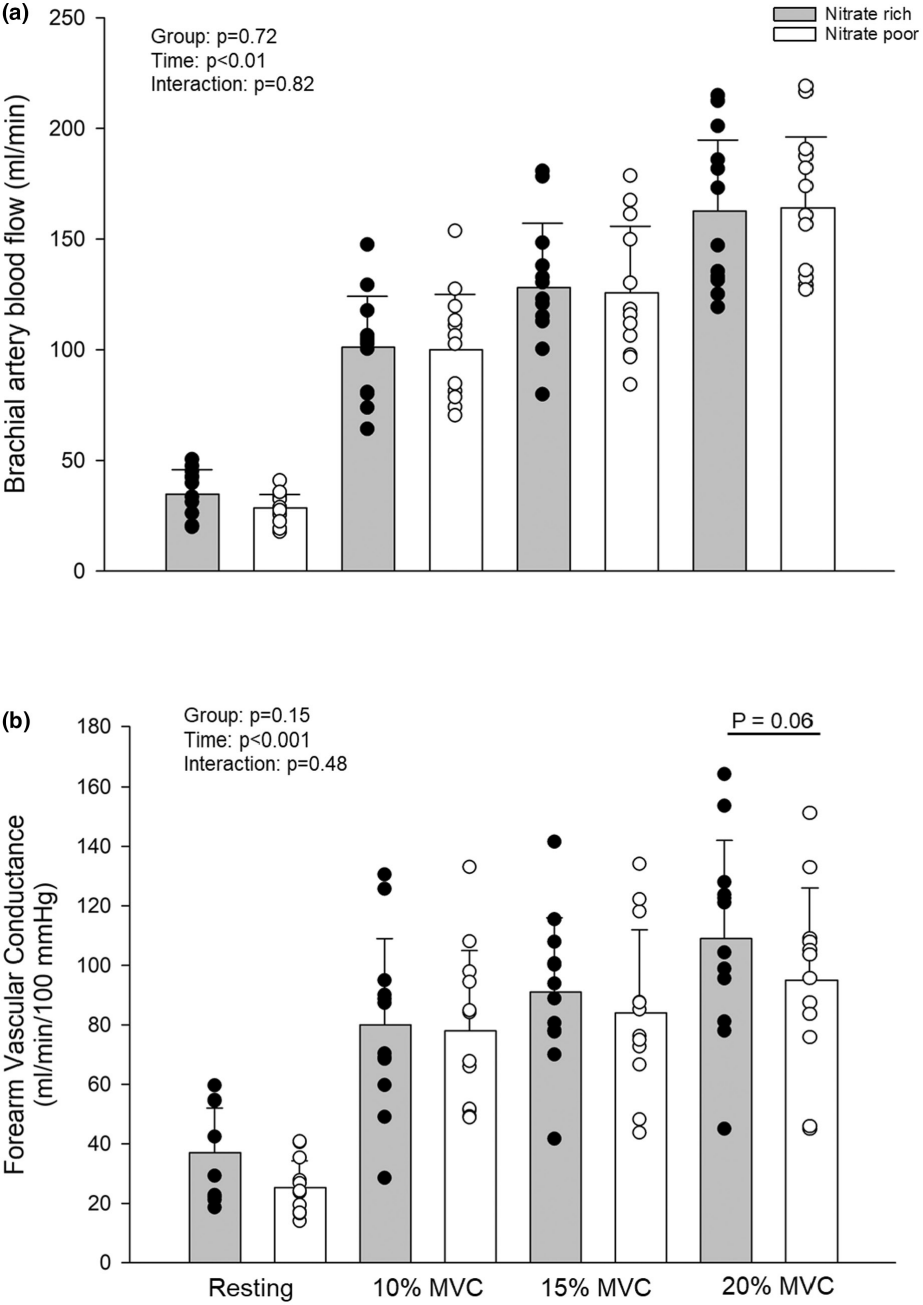
(a) No significant change to brachial artery blood flow responses to incremental handgrip exercise between nitrate rich (*n* = 12) or nitrate poor groups (*n* = 12) (*p* > 0.05). (b) No significant change in brachial artery vascular conductance to incremental handgrip exercise between nitrate rich (*n* = 12) or nitrate poor (*n* = 12) groups (*p* > 0.05). Data were run with two‐way ANOVA. Data are means ± SD.

Incremental exercise protocol showed no differences in FBF at any intensity during the NR or NP interventions. Similarly, vascular conductance was similar between treatments and intensities with 20% MVC showing *p* = 0.06 (Figure 6a,b). A significant treatment effect was shown with steady‐state MAP blood pressure responses being lower in all NR treatment conditions (10%, NR 120 ± 6, NP 132 ± 5; 15%, NR 124 ± 7, NP 141 ± 6; 20; 20%, NR 129 ± 6, NP 142 ± 5 mmHg) (all *p* < 0.05).

**FIGURE 7:**
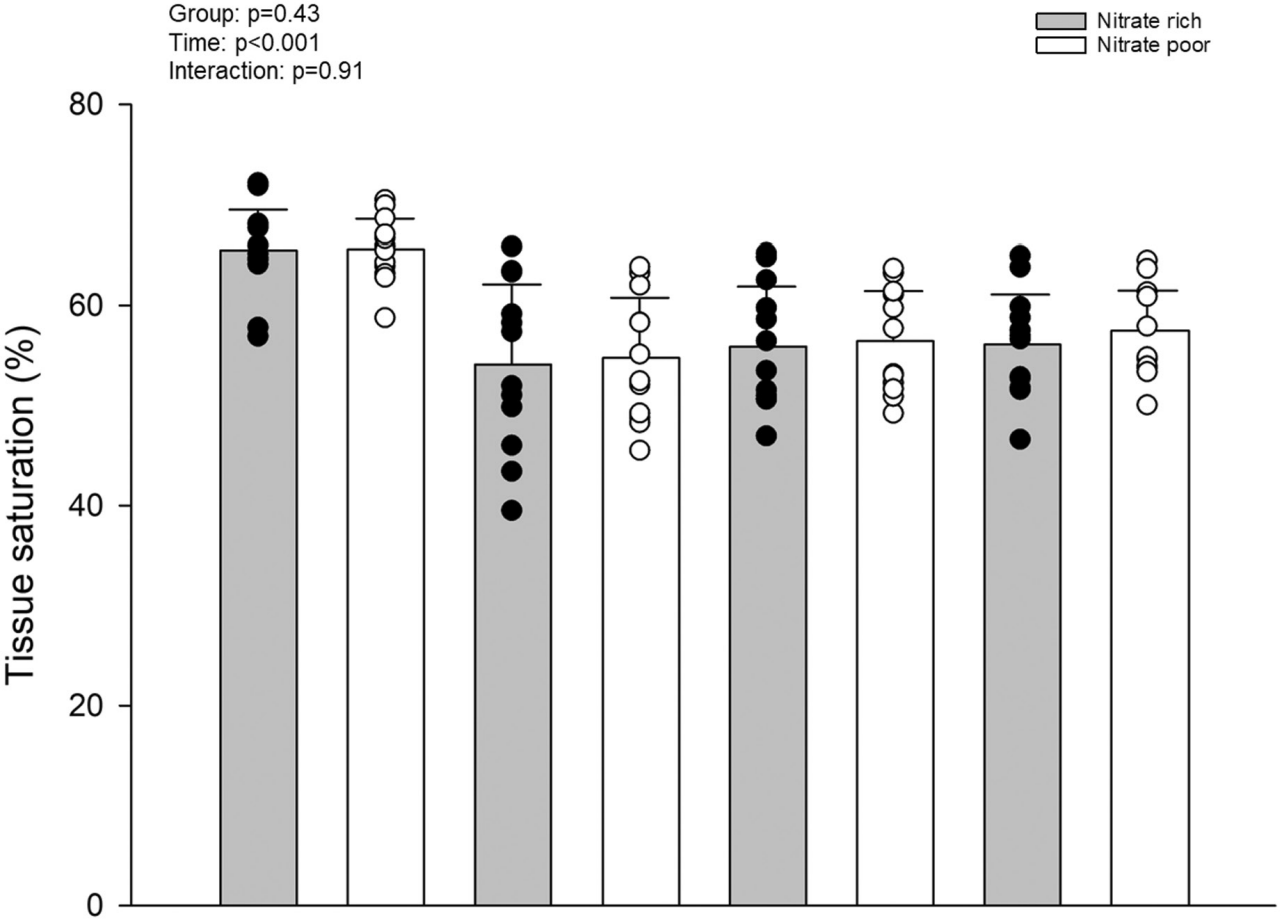
NIRS derived forearm tissue saturation during incremental handgrip exercise (*n* = 12 for both groups). No significant changes shown (*p* > 0.05). Data were run with two‐way ANOVA. Data are means ± SD.

The NIRS‐derived rate of oxygen desaturation was not different between groups during either the resting (NR, −0.13 ± 0.03; NP, −0.13 ± 0.007%/s) or ischemic exercise PORH (NR, −0.6 ± 0.2; NP, −0.4 ± 0.1) (both *p* > 0.05). Microvascular responsiveness during the traditional PORH was not faster with a NR supplement (NR, 1.48 ± 0.12; NP, 1.75 ± 0.15%/s) (*p* = 0.062). Similarly, the ischemic exercise PORH was not significantly different with the NR supplement (NR, 2.12 ± 0.32; NP, 1.56 ± 0.28%/s) (*p* = 0.13) (Figure 7). The AUC (i.e., metabolic accumulation (McLay, Fontana, et al., 2016)) was not different between during either test (*p* = 0.68).

Aortic and brachial SBP, pulse pressure, MAP, and SEVR were significantly improved with the NR supplement (Table 2) (all *p* < 0.05). Neither brachial nor aortic diastolic pressure was reduced in the NR condition. cfPWV was not significantly reduced with NR supplementation (Table 2). Finally, the reflected, but not forward, wave was faster with nitrate.

## 5 DISCUSSION

The aim of the present study was to examine vascular outcomes following acute inorganic dietary nitrate supplementation at rest, during ischemic exercise, and during incremental hand‐grip exercise in PMF. Our study confirms that acute nitrate supplementation does not enhance traditional FMD or NIRS‐derived microvascular reperfusion (Hogwood et al., 2023; Somani et al., 2022). Further, we show that adding ischemic exercise—a stimulus likely to facilitate the reduction of nitrite to NO—did not enhance brachial FMD or microvascular PORH in the NR condition (Casey et al., 2015). No increases in brachial artery blood flow, conductance, or forearm tissue saturation were seen during incremental hand‐grip exercise. However, we show that the NR group reduced mean arterial pressure during small muscle mass exercise at all intensities. Finally, our findings align with previous research, showing that acute nitrate supplementation in PMF effectively reduces both central (aortic) and peripheral (brachial) blood pressure without altering vascular stiffness (Hughes et al., 2016; Kim et al., 2019). These results suggest that acute NR supplementation reduces blood pressure at rest and during exercise, lowering cardiac workload, but does not improve vascular responses or vascular structure in late‐phase PMF.

### 5.1 Rest to exercise responses

Our hypothesis that resting vascular function would remain unchanged and ischemic exercise would enhance the vasodilatory response in the NR condition is partially supported by our data. Traditional brachial artery FMD and NIRS forearm microvascular responses were not increased in the NR condition, consistent with previous findings indicating that short‐term increases in nitrate do not enhance endothelial‐mediated vascular function in PMF (Hogwood et al., 2023; Somani et al., 2022). Although nitrate/nitrite levels were elevated in the NR condition, 3 min of ischemic exercise did not enhance brachial artery FMD, or forearm microvascular reperfusion compared to the NP control. This result was unexpected, given that acidic and hypoxic conditions typically promote the reduction of nitrite to NO (Casey et al., 2015; Ferguson et al., 2013b; Richards et al., 2018). Likewise, ischemia and ischemic exercise will influence other vasodilatory mechanisms beyond NO (e.g., potassium, prostaglandins, reduced oxygen availability, oxidative stress (Laughlin et al., 2012)), that could influence our results. It is also important to consider that the %FMD herein is on average higher than previous reports which suggests adequate endothelial function in our cohort (Hogwood et al., 2023; Moreau et al., 2013, 2020; Moreau & Ozemek, 2017). Reports also indicate high levels of oxidative stress within PMF that reduce the effectiveness of the NR supplement but given the range of FMD% this does not seem likely. These findings reinforce the need for trials using chronic supplementation to improve efficacy (Delgado Spicuzza et al., 2024), consider alternative vasodilatory mechanisms, and the level of FMD impairment in late‐phase PMF (Hogwood et al., 2023; Swift et al., 2014).

The present results show that acute NR supplement had no impact on brachial artery blood flow, conductance, or forearm saturation during incremental exercise. However, forearm vascular conductance was 13% higher in the NR condition at 20% MVC (*p* = 0.06; Cohen's *d* 0.53). Moreover, MAP during all phases of incremental exercise was lower with the NR supplement. This is important as previous work clearly shows that PMF have reductions in blood flow to the working muscle, accompanied by higher blood pressures, increased vascular resistance, and reductions in vasodilation (Mercuro et al., 2006; Moore et al., 2012; Moreau et al., 2003; Proctor et al., 2003). Our results did not show improvements in vasodilation, but lower resting and exercise blood pressure suggest systemic vascular resistance was likely reduced. As such, acute nitrate supplementation is useful for exercising blood pressure but requires future work to help elucidate our findings on endothelial health, local vasodilatory control (Fadel et al., 2004), and exercise performance.

### 5.2 Central and peripheral blood pressure changes

Our findings also support the efficacy of acute dietary nitrate supplementation in reducing both central and peripheral blood pressure parameters. Previous well‐controlled studies have demonstrated that acute nitrate supplementation decreases aortic systolic pressure, but not diastolic pressure or pulse pressure, in aged males and females (Hughes et al., 2016), and PMF (Kim et al., 2019). Our results corroborate these findings by showing reductions in both aortic and brachial systolic, but not diastolic, pressures with nitrate supplementation. This effect may be attributed to selective vasodilation of larger conduit arteries rather than resistance vessels (Kim et al., 2019; Omar et al., 2015) as evidenced by our NIRS data indicating that microvascular function was not affected by the nitrate supplement. It is interesting to note that we found no differences in brachial artery FMD responses between conditions. While this may indicate no change in conduit vasodilation, prior evidence clearly indicates intraarterial inorganic nitrite selectively dilates the radial artery (Omar et al., 2015). However, our results may be explained by the small vessel segment or area of interrogation measured with our techniques. Regardless, the observed reductions in central and peripheral systolic pressures are similar to pharmaceutical interventions (Kapil et al., 2014) and have significant clinical importance as these may result in reduced incidence of CV disease and events (Kapil et al., 2014, 2015; Lundberg et al., 2018).

It is well established that cfPWV is an independent predictor of CV risk (Kapil et al., 2014) as a 1 m/s increase is associated with a 17% and 13% increase in CV and all‐cause mortality, respectively (Vlachopoulos et al., 2019). Although our data did not show a statistically significant reduction in cfPWV, the observed decrease in central vascular stiffness remains clinically meaningful (Zocalo & Bia, 2021). This finding aligns with the previous studies by Hughes et al. (2016) and Kim et al. (2019), which also reported no effect of nitrate supplementation on vascular stiffness despite reductions in blood pressure. A notable factor to consider is the time since menopause, which was not detailed in previous studies. This is crucial as vascular aging varies significantly and is strongly influenced by hormonal status (Moreau et al., 2024; Moreau & Ozemek, 2017). In our study, the average time since menopause for participants was 16 ± 3 years, which may have implications for the outcomes in endothelial and pressure responses observed herein (Moreau et al., 2013, 2020, 2024; Moreau & Ozemek, 2017).

### 5.3 Experimental considerations

The present study used a randomized, double‐blind, crossover design without a true placebo condition which must be considered, though an increase in nitrate and nitrite following consumption of NR beetroot juice was shown. We did not control participants diets but did ask them not to change their diet, to refrain from mouthwash, and to be fasted for at least 4 h prior to visits. Some PMF had relatively good resting %FMD while some did not, and this range of responses likely impacted the power of the current study and must be considered. Moreover, our participants consumed the supplements prior to laboratory visits, which limited our ability to assess impaired FMD responses prior to supplementation. MVC was taken on the first day and may impact basal conditions, however given that this study was randomized, and baseline parameters for FMD and NIRS were similar we do not believe this impacted the study results. No oxidative stress measurements were taken and should be considered when interpreting our data. Finally, while it should not impact the outcome of a paired design, adipose tissue thickness was not assessed in every participant and was not included but must be acknowledged.

## 6 CONCLUSION

In summary, these data highlight the positive impact of acute dietary nitrate in PMF to reduce central and peripheral systolic pressures and improve hemodynamics. Further, we show lower mean arterial pressure at each exercise intensity and a trend for higher vascular conductance at 20% MVC, while the effect on endothelial and microvascular function was not apparent. Our data coupled with existing evidence suggests that long‐term nitrate treatment is likely needed to optimize vascular control and when combined with higher intensity exercise may to improve the vascular environment and lower CV disease risk without the use of pharmacology.

## AUTHOR CONTRIBUTIONS

J. T .C., S. J. J., and S. A. F. conceived and designed research; J. T. C., A. K., E. E. G., M. E. S., and L. Z. performed experiments; J. T. C., and S. J. J., E. E. G., M. E. S. J. D. A analyzed data; J. T. C., A. K., L. Z., and S. J. J interpreted results of experiments; J. T. C., S. A. F., and A. K. prepared figures; J. T. C., A. K., and S. J. J., E. E. G., M. E. S. J. D. A drafted manuscript; J. T. C. A. K., L. Z., S. A. F., and S. J. J., E. E. G., M. E. S. J. D. A edited and revised manuscript; J. T. C. and S. J. J approved final version of manuscript.

## FUNDING INFORMATION

This work was supported by the University of Wisconsin‐La Crosse Research Service and Educational Leadership award won by Alyssa Koenke.

## CONFLICT OF INTEREST STATEMENT

The authors declare no conflicts of interests.

## ETHICS STATEMENT

This study was approved by the University of Wisconsin La‐Crosse Institutional Review Board (23‐JC‐40) and has therefore been performed in accordance with the ethical standards in the Declaration of Helsinki.

## Data Availability

Data will be made available upon reasonable request.
